# The archaeal class *Halobacteria* and astrobiology: Knowledge gaps and research opportunities

**DOI:** 10.3389/fmicb.2022.1023625

**Published:** 2022-10-13

**Authors:** Jia-Hui Wu, Terry J. McGenity, Petra Rettberg, Marta F. Simões, Wen-Jun Li, André Antunes

**Affiliations:** ^1^State Key Laboratory of Lunar and Planetary Sciences, Macau University of Science and Technology (MUST), Taipa, Macau SAR, China; ^2^China National Space Administration (CNSA), Macau Center for Space Exploration and Science, Taipa, Macau SAR, China; ^3^School of Life Sciences, University of Essex, Colchester, United Kingdom; ^4^German Aerospace Center (DLR), Institute of Aerospace Medicine, Köln, Germany; ^5^State Key Laboratory of Biocontrol, Guangdong Provincial Key Laboratory of Plant Resources and Southern Marine Science and Engineering Guangdong Laboratory (Zhuhai), School of Life Sciences, Sun Yat-sen University, Guangzhou, China

**Keywords:** astrobiology, archaea, *Halobacteria*, extremophiles, Mars, icy moons

## Abstract

Water bodies on Mars and the icy moons of the outer solar system are now recognized as likely being associated with high levels of salt. Therefore, the study of high salinity environments and their inhabitants has become increasingly relevant for Astrobiology. Members of the archaeal class *Halobacteria* are the most successful microbial group living in hypersaline conditions and are recognized as key model organisms for exposure experiments. Despite this, data for the class is uneven across taxa and widely dispersed across the literature, which has made it difficult to properly assess the potential for species of *Halobacteria* to survive under the polyextreme conditions found beyond Earth. Here we provide an overview of published data on astrobiology-linked exposure experiments performed with members of the *Halobacteria*, identifying clear knowledge gaps and research opportunities.

## Introduction

Astrobiology is a relatively recent but increasingly relevant cross-disciplinary field of research, which focuses on the origin, distribution, and evolution of Life in the universe (Des Marais et al., 2008; Horneck et al., 2016; Hallsworth et al., 2021b). Fittingly for such a wide scope of study, astrobiology combines techniques and expertise from fields as diverse as biology, chemistry, geology, or planetary sciences (Dartnell and Burchell, 2009; Race et al., 2012; Taşkın and Aydinoglu, 2015). With the increase in frequency and broadening of scope of space exploration, including human space travel, it has become vital to better understand the survival and reproduction of microbial life under multiple extreme conditions, and their impact on space exploration missions and on human travelers who they may encounter or with whom they may cohabit (Cavicchioli, 2002; Moissl-Eichinger, 2011; Moissl-Eichinger et al., 2016; Cottin et al., 2017; Checinska Sielaff et al., 2019; Merino et al., 2019; Nickerson et al., 2022).

### Terrestrial analogs

One of the main pillars of astrobiology is the investigation of specific terrestrial analog environments and the study of their microbial inhabitants. Such analogs exhibit characteristics found in other locations in the Solar System (Preston and Dartnell, 2014; Martins et al., 2017). Thus far, most terrestrial analog sites used for astrobiology have been selected based on conditions found on Mars (Fairén et al., 2010; Martins et al., 2017), although a few suitable analogs for the exooceans of the icy moons of the outer solar system have also been proposed (Marion et al., 2003; Kereszturi and Keszthelyi, 2013; Antunes et al., 2020).

The study of terrestrial analog sites is seen as essential for: (i) studying the limits of life (e.g., Azua-Bustos et al., 2012; DasSarma et al., 2017; Koschnitzki et al., 2021), (ii) obtaining new microbes for astrobiological exposure experiments (e.g., Musilova et al., 2015; Beblo-Vranesevic et al., 2020), (iii) analyzing long-term viability and preservation of microbes and biomolecules (e.g., Gramain et al., 2011; Leuko et al., 2017; Schreder-Gomes et al., 2022), (iv) technology development and testing for life-detection in space missions (e.g., Harris et al., 2015; García-Descalzo et al., 2019; Lantz et al., 2020; Dypvik et al., 2021; Rull et al., 2022), and (v) defining and refining planetary protection measures (e.g., Rettberg et al., 2019).

When analyzing the potential viability of microbes in other parts of our Solar System, several environmental factors need to be taken into consideration (Horneck et al., 2010; Moissl-Eichinger et al., 2016; Cottin et al., 2017). The limited information on the physical-chemical conditions present in the exooceans of the icy moons partly restrains more in-depth analyses on the survivability of microbes, despite some very interesting studies on potential microbial metabolisms (e.g., Taubner et al., 2018; Goordial, 2021; Sherwood Lollar et al., 2021). This is in stark contrast with the large amount of information already available about Mars and its apparently unwelcoming conditions. These are particularly harsh to mesophiles, combining high levels of radiation (particularly ultra-violet UVC and UVB) (Cockell et al., 2000; Vicente-Retortillo et al., 2020; Zhang et al., 2022), desiccation/low water activity (Knoll and Grotzinger, 2006; Stevenson et al., 2015b; Toner and Catling, 2018; Rivera-Valentín et al., 2020; Hallsworth et al., 2021a), high concentrations of salts and volatile oxidants induced by radiation (Quinn et al., 2013; Lasne et al., 2016; Wu et al., 2018), locations with acidic conditions (Benison and Bowen, 2006; Bibring et al., 2006; Noe Dobrea and Swayze, 2010; Ehlmann et al., 2016), solar particle events (Lillis and Brain, 2013; Ramstad et al., 2018), low temperatures, low pressures (Pätzold et al., 2016; Spiga et al., 2018), and low nutrient availability (Tomkins et al., 2019; Fackrell et al., 2021; Tarnas et al., 2021). Although the extreme setting of Mars has traditionally been seen as biocidal, this is not necessarily the case, as recently highlighted by Hallsworth (2021).

Water is essential for the existence and development of all known life forms, so the search for life in other parts of our solar system and beyond is directly linked with the existence of this vital substance. Luckily for our pursuits, water seems to be more abundant than previously thought (Nimmo and Pappalardo, 2016; Grasset et al., 2017; Greenwood et al., 2018; Schörghofer et al., 2021); there is now evidence that water deposits exist on Mars (Ojha et al., 2014; Martín-Torres et al., 2015; Stillman et al., 2017; Rivera-Valentín et al., 2020; Lauro et al., 2021; Deutsch et al., 2022) as well as on several icy moons (Spencer and Nimmo, 2013; Vance et al., 2014; Hayes, 2016; Jia et al., 2018; Genova et al., 2022). Despite several unknowns, these water bodies outside our planet appear to contain high levels of salt, including perchlorate (Navarro-González et al., 2010; Oren et al., 2014; Martín-Torres et al., 2015; Lauro et al., 2021). Therefore, the study of Earth’s wide range of high salinity environments (from salterns, to polar brine aquifers, deep-sea brines or halite and other salt deposits), as well as their microbial inhabitants, has become increasingly relevant in the context of astrobiology.

### Halophiles as models for astrobiology

Organisms living in high salinity environments are known as halophiles (i.e., salt loving), with representatives spread across the three domains of Life. Microbes living specifically at the higher range of salinities are almost exclusively composed of members of the archaeal class *Halobacteria*, a phylogenetically coherent group of extreme halophiles (Edbeib et al., 2016; DasSarma and DasSarma, 2017). Among other properties, members of this class are known for their resilience and ability to thrive under multiple environmental extremes (polyextremophilic) including high salinity, high levels of radiation, vacuum, extreme cold, utilization or growth in the presence of caustic materials such as perchlorate, oxygen deprivation, low-nutrient availability, and desiccation, all of which are relevant for astrobiology and particularly important in a Martian context (e.g., Rittmann et al., 2004; McCready et al., 2005; Crowley et al., 2006; Baxter et al., 2007; Coker et al., 2007; Oren et al., 2014; Stan-Lotter and Fendrihan, 2015; Stevenson et al., 2015a; Laye et al., 2017; Sorokin et al., 2017; Thombre R. et al., 2017; Laye and DasSarma, 2018; Leuko et al., 2020; further details provided in Section “The archaeal class Halobacteria and astrobiology: Astrobiological testing,” and Table 1).

**TABLE 1:** Overview of *Halobacteria* tested for astrobiology-relevant features (number of species/total species, genera/total genera in each taxon).

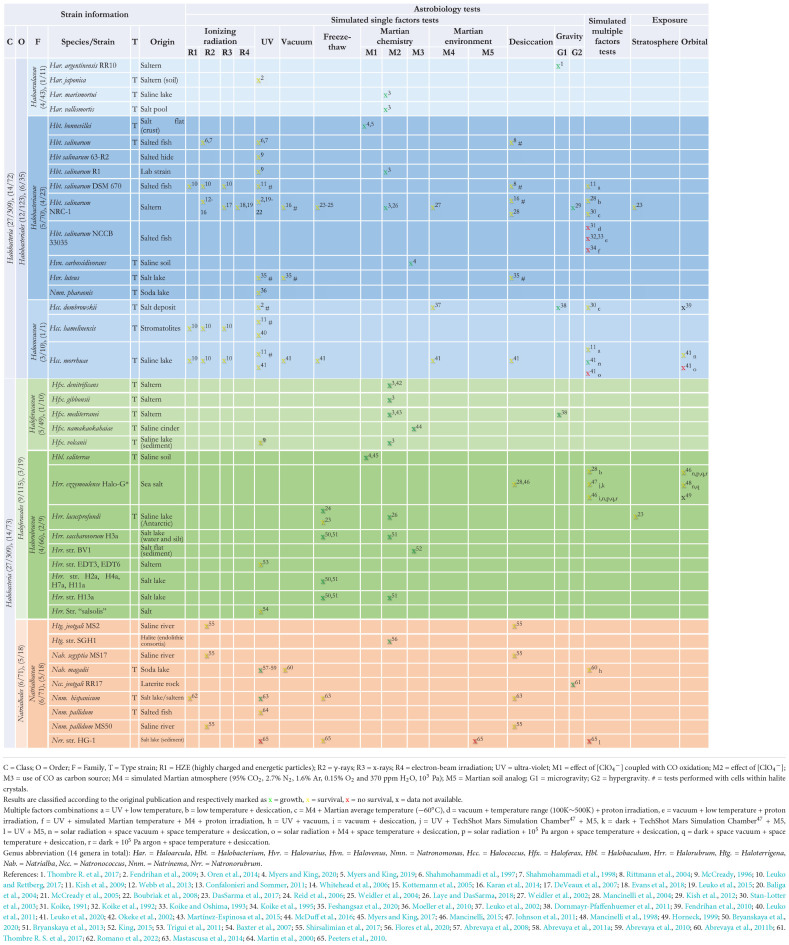

As a result of their unique combination of adaptations and capabilities, these polyextremophilic microbes have long been hailed as models for astrobiology and flagged as priority groups for testing (DasSarma, 2006; Reid et al., 2006; Baxter et al., 2007; DasSarma et al., 2020). Accordingly, several authors have followed this research thread and data has been accumulating. However, a systematic approach for thoroughly testing *Halobacteria* or a good overview of its current status seems to be missing. We aimed to help to address this growing issue by collecting all available data from primary literature on exposure-based testing with strains of this group, mapping the results as reported in their original publications, and discussing the snapshot that we have recorded.

## The archaeal class *Halobacteria* and astrobiology: Taxonomic diversity

Currently, the class *Halobacteria* is grouped into six families and three orders (Gupta et al., 2016). The order *Halobacteriales* presently has the highest number of species within the group and is split into three families: *Haloarculaceae*, *Halobacteriaceae*, and *Halococcaceae*. *Halobacteriaceae* is the largest family within the *Halobacteria*, contrasting with the *Halococcaceae* which is the smallest one and comprises a single genus (and only 10 species). To date, the order containing the second-highest number of species is *Haloferacales*, spread across two families: *Haloferacaceae*, and *Halorubraceae*, followed by the order *Natrialbales*, which includes the single family *Natrialbaceae*.

As outlined in Table 1 and in Supplementary Figure 1, only a relatively small number of species within the class *Halobacteria* has been tested for their capacity to grow or survive under astrobiological- or Martian-relevant conditions, with a slightly better performance when looking at genus-based coverage. This is reflected both when looking at numbers for each class and when breaking down into intermediate taxa level such as order or family. The tested species are usually below 10% of the total number within each taxon, and between 10 and 20% for tested genera. The most noteworthy exception to these ranges can be found within the *Halococcaceae*, as it is composed of a single genus and has a small number of total species. These low numbers occur despite the widely recognized importance of the *Halobacteria* for astrobiology (e.g., Kunte et al., 2002; DasSarma, 2006; Reid et al., 2006; Baxter et al., 2007; Leuko et al., 2014; Oren, 2014a; Rettberg et al., 2019; DasSarma et al., 2020).

Another aspect worth highlighting is that most astrobiology-based studies have focused on a very narrow range of genera and species within the class *Halobacteria*, mostly associated with members of the order *Halobacteriales*, and particularly focused on the genus *Halobacterium* (Table 1). Indeed, the *Halobacteriales* represent more than half of the tested species within the class, followed by the *Haloferacales* (nine tested species), and by the almost unexplored *Natrialbales* (six species). Furthermore, within several families of the class we find a relatively common trend: tests are usually focused on a single genus (Table 1). This can be seen in the order *Halobacteriales* for family *Haloarculaceae* (genus *Haloarcula*) and family *Halococcaceae* (genus *Halococcus*), but also in the order *Haloferacales* for the family *Haloferacaceae* (genus *Haloferax*).

A better representation of diversity is found within the family *Halobacteriaceae* with four different genera (*Halobacterium*, *Halovarius*, *Halovenus*, and *Natronomonas*), despite the clear emphasis on *Halobacterium*.

Regarding the strains selected for astrobiology-related testing, most of them are type strains for their respective species (Table 1). The use of such type strains is usually recommended, as this contributes to more readily available data on the strains, and their accessibility facilitates experimental reproducibility and comparative studies (Antunes et al., 2017). One very clear and notable exception to this predominance of type strains is the use of *Halobacterium* sp. NRC-1. Although not a type strain, it has been deposited in microbial biological resource centers and is readily available; it has also been very extensively studied and has long been considered a model system for studies in Archaea, namely due to ease of culturing and genetic manipulation (Ng et al., 2000; McCready et al., 2005; Boubriak et al., 2008; Anderson et al., 2016; Dassarma et al., 2016). The status of this strain explains the very extensive number of astrobiological-based tests performed with it (Table 1).

## The archaeal class *Halobacteria* and astrobiology: Astrobiological testing

Another insight of our analysis is the realization of how restricted and limited datasets on testing currently are. Indeed, most exposure experiments performed with the class *Halobacteria* focus exclusively on the effects of a single environmental factor (with a noticeable predominance of radiation-exposure tests; Table 1 and Supplementary Figure 2). While the testing of one variable at a time is consistent with classical experimental techniques, the low number of tests with multiple variables is limiting. This gap in our understanding of the effect of simultaneous exposure to multiple extreme conditions is particularly apparent given that astrobiology-relevant settings beyond our planet include a combination of multiple environmental extremes (e.g., Merino et al., 2019; Simões and Antunes, 2021).

Space-exposure testing provides the ideal platform for obtaining a better perspective on this, but the number of tested *Halobacteria* species remains low as most experiments have made use of simulated conditions only. However, it should be noted that these issues are not exclusive to *Halobacteria* but are rather major general limitations that apply to most microbial taxa. Several upcoming projects and recent publications have flagged members of the *Halobacteria* as key target organisms (Sgambati et al., 2020; Beblo-Vranesevic et al., 2022), so we can expect relevant new results in the near future.

Despite the generalized sparsity of data across the board, we have some good examples worth highlighting regarding completeness of the datasets. It should be relatively easy to complete a few datasets and cover some of the most obvious gaps. For example, testing the effects of exposure to UV currently has been performed for at least one representative of each family of the class *Halobacteria* except for the *Halorubraceae* (Table 1 and Supplementary Figure 2). Likewise, the family *Haloarculaceae* is currently the only one that has not been subjected to exposure to multiple combined extremes (Table 1 and Supplementary Figure 2).

Looking at the individual species/strain level (Table 1), we also have some good examples that are close to complete. *Halobacterium salinarum* NRC-1 has the most complete datasets, with very few gaps (12 out of 18 listed astrobiology tests). As previously mentioned, the latter is widely recognized as a model organism within the *Halobacteria* and an extensive amount of testing has been done with this strain. Nonetheless, regarding simulated single-factor astrobiology tests, to date there are no reported results for exposure to microgravity and several Martian chemistry and environment factors (M1 and M3–M5; Table 1 and Supplementary Figure 2). Furthermore, this strain has been included in stratospheric exposure experiments, but has not been subjected to orbital testing. Given its status as model organism (McCready et al., 2005; Boubriak et al., 2008; Dassarma et al., 2016), this strain should be flagged as a priority for future tests.

The second good example we would like to highlight regarding completeness is *Halococcus morrhuae*. Although currently having less complete datasets than *Halobaculum salinarum* NRC-1 (10 out of 18 listed astrobiology tests; Table 1), it could be flagged as a further target for future completion. This species has the advantage of having already been used in orbital exposure tests, which are arguably the most challenging from a logistic and access point of view.

*Natriabales* is the only order of *Halobacteria* that does not have a representative already subjected to orbital testing. This order has the most incomplete datasets, as it is also absent from testing for most radiation types (with the notable exception of UV), most Martian chemistry and environmental factors (apart from testing with Martian soil analogs), desiccation, microgravity, and stratospheric testing (Table 1 and Supplementary Figure 2).

Other important factors, not included on the overview table are exposure to low temperatures and low pH. Both conditions are seen as relevant from a Martian perspective but have not been directly listed as both are part of the standard characterization of new species. The number of species of *Halobacteria* capable of growth below 15°C or below pH 5 is notably small (currently consisting of 3 and 9 species, respectively). Nonetheless, within those, only *Halorubrum lacusprofundi* has been targeted for astrobiology testing.

On Earth, sites which are simultaneously acidic and highly saline are relatively rare (Mormile et al., 2009; Conner and Benison, 2013; Escudero et al., 2018), and studies on cold hypersaline sites remain largely underexplored (Oren, 2014b; Lamarche-Gagnon et al., 2015; Sapers et al., 2017). This might explain the reduced number of species within the *Halobacteria* that could be highlighted here. Further efforts in exploring these sites should be prioritized and could prove quite beneficial.

Looking at coverage based on the type of stressor also uncovered some unexpected areas where further work needs to be done (Supplementary Figure 2). Selected highlights of these gaps include exposure to single factors such as microgravity (missing data for *Halobacteriaceae*, *Halorubraceae*, and *Natrialbaceae*), simulated Martian atmosphere (with no representation for *Natrialbales*, *Haloferacales*, or *Haloarculaceae*) or Martian soil analogs/regolith (only tested for the *Natrialbaceae*). On the more complete datasets, tests looking at exposure to desiccation or to perchlorate are still needed to ensure that all families are represented.

## Future work: Knowledge gaps and research opportunities

As outlined above, we can see that there are currently clear and significant gaps in the study of the class *Halobacteria* under the scope of Astrobiology. More in-depth testing is needed to cover the taxonomic diversity present in this archaeal group both at higher- and lower-ranking taxonomy levels (particularly in families with single-genus testing or with few tested species). The fact that species of this class have large pangenomes, such that even closely related taxa may be functionally quite distinct (Gaba et al., 2020; de la Haba et al., 2021), further emphasizes the need for more extensive testing.

Likewise, there is a need for more completed datasets on astrobiology-relevant stressors on members of this class, both as single factors and combined, particularly making use of space-exposure experiments. A notable (and surprising) gap here is on the study of the effects of different salts. Although their relationship with salt is at the core of the definition of halophiles, studies have been almost exclusively restricted to sodium chloride (NaCl). The astrobiological relevance of studying the interaction with different salts (including perchlorates), as well as looking into chaotropic and kosmotropic effects, is clear and has been well noted (Hallsworth et al., 2007; Ball and Hallsworth, 2015; Stevenson et al., 2015b; dC Rubin et al., 2017). Yet, most species of *Halobacteria* still remain untested. Another noticeably neglected field here is the study of exposure to high pressure. Although technically more challenging, these settings would be very relevant in the context of the Martian subsurface or of the exooceans of the icy moons (Ueta and Sasaki, 2013; Jebbar et al., 2020; Olson et al., 2020).

From a physiological diversity perspective, new opportunities might also be opening up. Although members of the *Halobacteria* were typically seen as aerobic heterotrophs (Andrei et al., 2012) and reliant on the “salt-in” strategy (e.g., Kunte et al., 2002), they have consistently proven to be a lot more diverse (Müller et al., 2005; Oren, 2008; Gunde-Cimerman et al., 2018). An increasing number of species has been reported to use alternative anaerobic metabolic pathways, or even to show preference for anaerobic conditions or phototrophy (e.g., Antunes et al., 2008; Sorokin et al., 2017, 2018; Weber et al., 2017; Miralles-Robledillo et al., 2021).

There might be possible implications of this versatility in overall resilience and adaptability to astrobiology-relevant conditions. Microorganisms with “less typical” features could thus be interesting candidates for targeted future exposure studies and comparisons. An over-reliance on quickly growing or easier strains in detriment of those with higher relevance for astrobiology, such as anaerobic ones (Beblo-Vranesevic et al., 2020) or psychrotolerant/psychrophilic (Kish et al., 2012), should also be avoided despite the additional challenges and technical issues they might bring (Hamm et al., 2019; Cui and Dyall-Smith, 2021; Wang et al., 2021).

From an ecological perspective, it is relevant to note that the vast majority of tested species were not isolated from environments traditionally, or officially, flagged as terrestrial analog sites, despite some notable exceptions (e.g., *Hrr. lacusprofundi*). It is true that the identification of terrestrial analog sites can be seen as somewhat subjective and, in some ways, all hypersaline sites could potentially be seen as Martian analog sites (e.g., Mancinelli et al., 2004; Parro et al., 2011; Murray et al., 2012; Xiao et al., 2017). Nonetheless, it would be a useful priority to place further stress on: (i) testing members of the class *Halobacteria* from well-established analog sites and (ii) further identification and flagging of additional hypersaline sites as suitable terrestrial analogs. Likewise, the increase in cultivation-based studies specifically aimed toward isolating new strains and describing new species from such sites, namely polar, deep-sea, or magnesium-sulfate brines (Franzmann et al., 1988; Antunes et al., 2008; Albuquerque et al., 2012; Mou et al., 2012), would also prove beneficial and provide excellent new targets for exposure tests (DasSarma et al., 2017; Laye and DasSarma, 2018).

Furthermore, while single-species experiments are valuable, we should note that microbes do not generally live in isolation (e.g., Van Der Wielen and Heijs, 2007; Vikram et al., 2016; Abed et al., 2018; Uritskiy et al., 2021). Therefore, parallel experiments involving constructed consortia (as well as characterized natural consortia) of halophiles, could provide a useful complementary approach (e.g., Wadsworth et al., 2019; Leuko et al., 2020). Other approaches could (and should) focus on the distinction between survival and growth/multiplication in exposure experiments or the issue of long-term viability of microbes trapped in salt, a very relevant topic for halophilic research (Gramain et al., 2011; Jaakkola et al., 2014; Stan-Lotter and Fendrihan, 2015), but still under-explored from an astrobiological perspective.

Access to launching opportunities has been rather restricted in the past and has severely limited capability to perform experiments in space, but this sector is rapidly changing. The on-going rise of the space private sector, the increased use of micro-satellites, or even the recent launch of the Chinese Space Station, all provide further launch and testing opportunities that should be harnessed to address at least some of the gaps that we have identified. Perhaps there is also room for technical improvements on reducing some historical resistance toward high-salt experiments in space (due to increased risks of corrosion and clogging), allowing for, e.g., the testing of more live strains in liquid medium rather than just in lyophilized, freeze-dried form (Barravecchia et al., 2018; Rcheulishvili et al., 2020).

Finally, we highlight the increasing relevance of data and its analysis in the context of *Halobacteria* and astrobiology. Obviously, the selection and identification of model organisms for astrobiology should make use of a variety of data on their capabilities and characteristics. The increased relevance of the class *Halobacteria*, together with its significant on-going increase in the description of new species and genera, as well as in genomic and phenotypic data (e.g., DasSarma et al., 2010, 2019; Oren, 2014b; Antunes et al., 2017; Cui and Dyall-Smith, 2021; Lach et al., 2021), make it notoriously difficult to navigate this ocean of relevant yet dispersed information. Despite some efforts targeting the collection of data from this specific taxon (e.g., Pfeiffer et al., 2008; DasSarma et al., 2010; Ukani et al., 2011; Sharma et al., 2014; Loukas et al., 2018; Reimer et al., 2019), the absence of dedicated tools to quickly compare species or even to quickly identify strains with a combination of selected properties can be seen as a significant bottleneck in a range of fields of applied research with this group of archaea. Further community-wide efforts and the development of such tools should be thus seen as a key priority for researchers working with this taxon in the context of astrobiology and beyond.

## Data availability statement

The original contributions presented in this study are included in the article/Supplementary material, further inquiries can be directed to the corresponding author.

## Author contributions

AA conceived this study. J-HW conducted the data collection and processing. J-HW and AA conducted the core analysis and interpretation of the data and wrote the manuscript. All authors contributed to the analysis and interpretation of the data, revised the manuscript, and have read and approved the final manuscript.

## Supplementary material

Supplementary Figure 1 Proportion of species and genera subjected to testing of astrobiology-relevant features within the class *Halobacteria* and each of its orders and families (see Table 1 for full information).

Supplementary Figure 2 Overview of total number of reports for different types of tests of astrobiological relevance performed for each family of the *Halobacteria* (see Table 1 for full information). R1, HZE (highly-charged and energetic particles); R2, γ-rays; R3, x-rays; R4, electron-beam irradiation; UV, ultra-violet radiation; vac, vacuum; temp, low temperature; M1, effect of ClO4^–^ coupled with CO oxidation; M2, effect of ClO4^–^; M3, use of CO as carbon source; M4, simulated Martian atmosphere (95% CO2, 2.7% N2, 1.6% Ar, 0.15% O2, and 370 ppm H_2_O, 10^3^ Pa); M5, Martian soil analogue; des, desiccation; G1, microgravity; G2, hypergravity; mult, multiple conditions; str, stratosphere testing; orb, orbital testing.
