# Supplementary figures and images for: The archaeal class Halobacteria and astrobiology: Knowledge gaps and research opportunities
Source: Front Microbiol. 2022 Oct 13;13:1023625. doi: 10.3389/fmicb.2022.1023625 (PMC9608585; doi:10.3389/fmicb.2022.1023625)

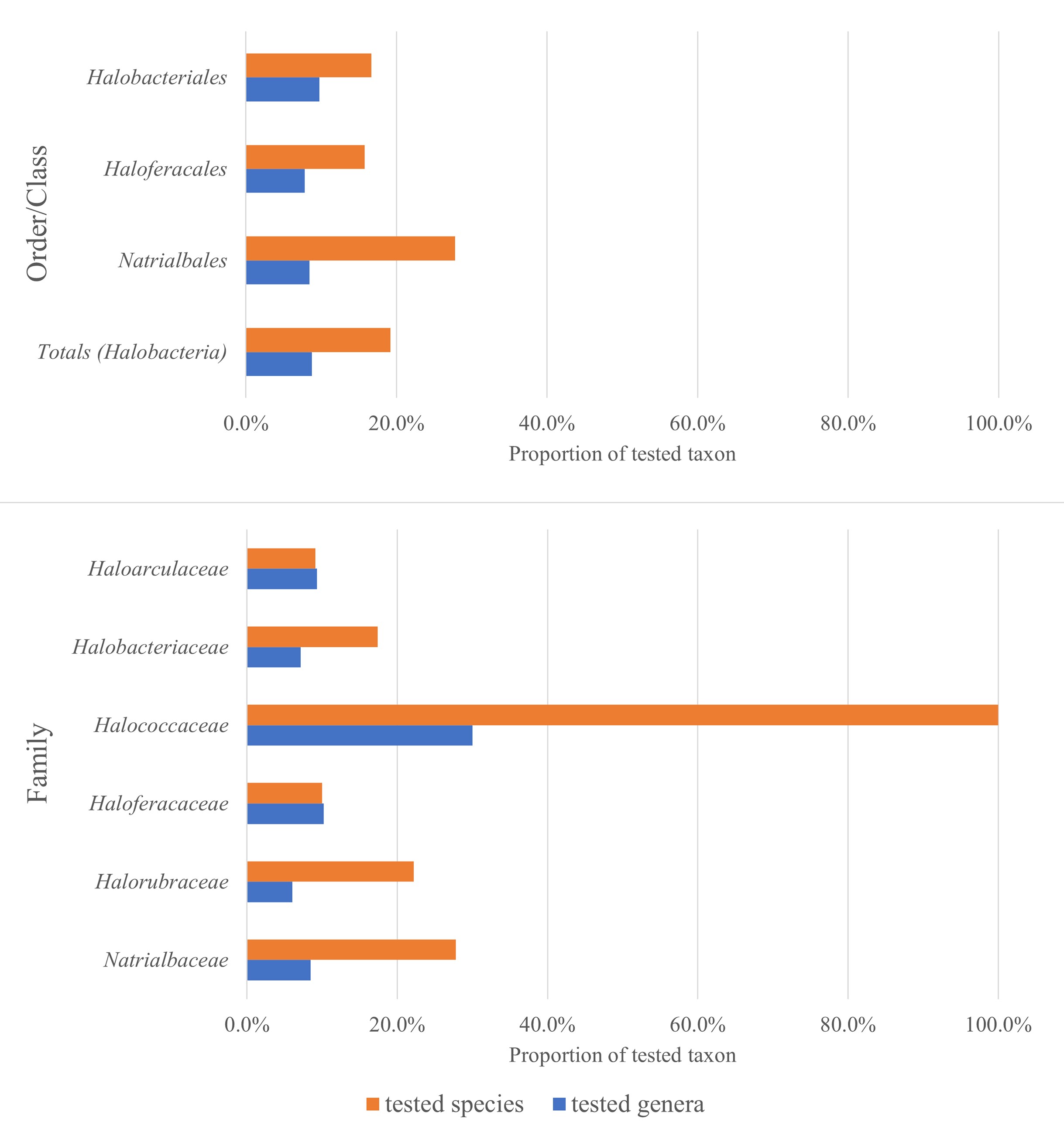

Supplement: Supplementary Figure 1 — Proportion of species and genera subjected to testing of astrobiology-relevant features within the class Halobacteria and each of its orders and families (see Table 1 for full information). [file Image_1.jpeg]

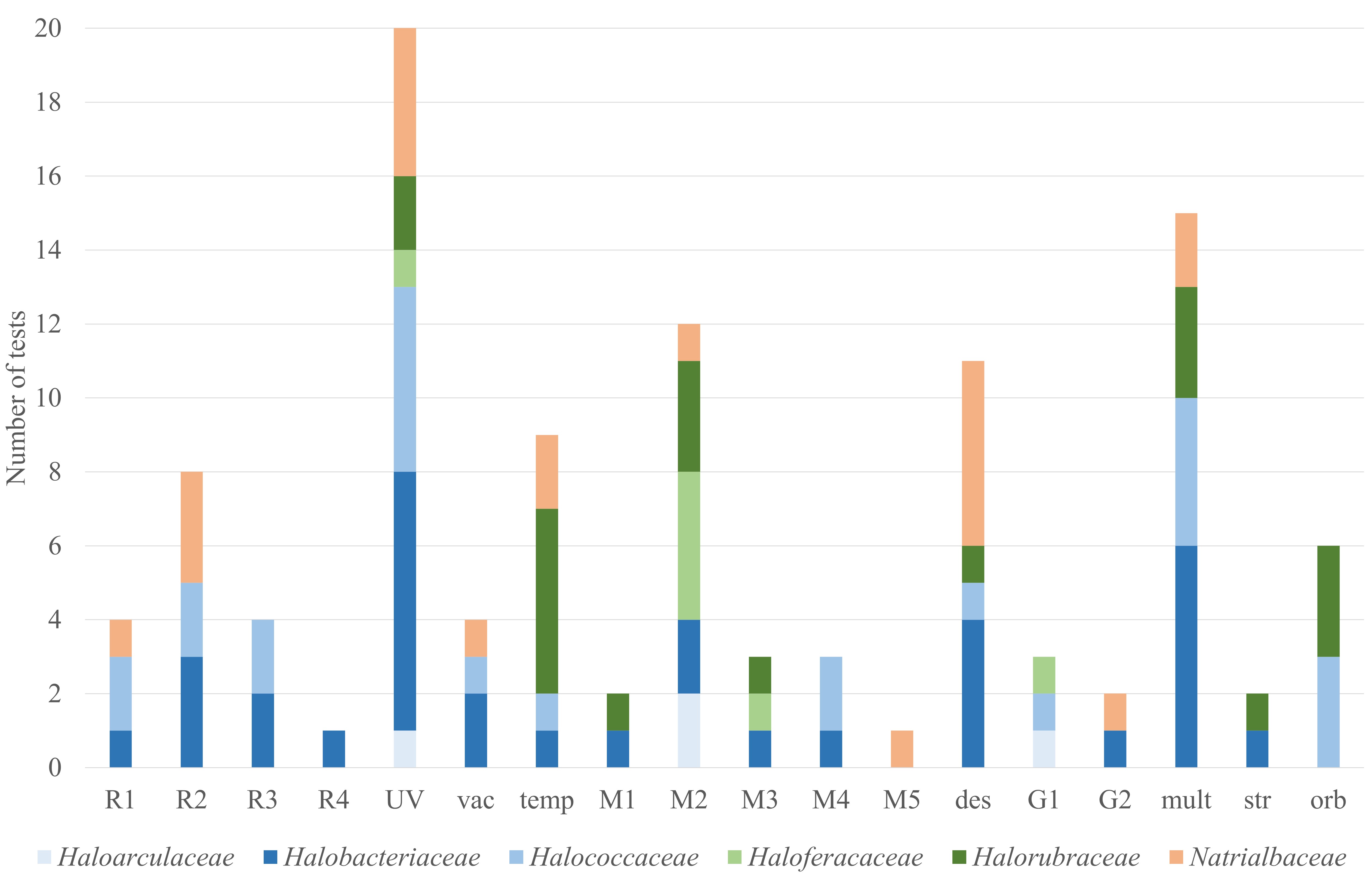

Supplement: Supplementary Figure 2 — Overview of total number of reports for different types of tests of astrobiological relevance performed for each family of the Halobacteria (see Table 1 for full information). R1, HZE (highly-charged and energetic particles); R2, γ-rays; R3, x-rays; R4, electron-beam irradiation; UV, ultra-violet radiation; vac, vacuum; temp, low temperature; M1, effect of ClO4– coupled with CO oxidation; M2, effect of ClO4–; M3, use of CO as carbon source; M4, simulated Martian atmosphere (95% CO2, 2.7% N2, 1.6% Ar, 0.15% O2, and 370 ppm H2O, 103 Pa); M5, Martian soil analogue; des, desiccation; G1, microgravity; G2, hypergravity; mult, multiple conditions; str, stratosphere testing; orb, orbital testing. [file Image_2.jpeg]
